# External Validation and Calibration of the DecaPreT Prediction Model for Decannulation in Patients with Acquired Brain Injury

**DOI:** 10.3390/brainsci11060799

**Published:** 2021-06-17

**Authors:** Elio Leto, Danilo Lofaro, Lucia Francesca Lucca, Maria Ursino, Stefania Rogano, Paolo Scola, Paolo Tonin, Domenico Conforti, Antonio Cerasa

**Affiliations:** 1 S. Anna Institute and Research in Advanced Neurorehabilitation (RAN), 88900 Crotone, Italy; e.leto@istitutosantanna.it (E.L.); l.lucca@istitutosantanna.it (L.F.L.); m.ursino@istitutosantanna.it (M.U.); s.roganoa@istitutosantanna.it (S.R.); p.scola@isakr.it (P.S.); p.tonin@isakr.it (P.T.); antonio.cerasa76@gmail.com (A.C.); 2 de-Health Lab—DIMEG, UNICAL, 87100 Arcavacata di Rende, Italy; domenico.conforti@unical.it; 3 Institute for Biomedical Research and Innovation (IRIB), National Research Council of Italy, 87050 Mangone, Italy

**Keywords:** acquired brain injury, tracheostomy, decannulation, prediction

## Abstract

We propose a new set of clinical variables for a more accurate early prediction of safe decannulation in patients with severe acquired brain injury (ABI), during a post-acute rehabilitation course. Starting from the already validated DecaPreT scale, we tested the accuracy of new logistic regression models where the coefficients of the original predictors were reestimated. Patients with tracheostomy were retrospectively selected from the database of the neurorehabilitation unit at the S. Anna Institute of Crotone, Italy. New potential predictors of decannulation were screened from variables collected on admission during clinical examination, including (a) age at injury, (b) coma recovery scale-revised (CRS-r) scores, and c) length of ICU period. Of 273 patients with ABI (mean age 53.01 years; 34% female; median DecaPreT = 0.61), 61.5% were safely decannulated before discharge. In the validation phase, the linear logistic prediction model, created with the new multivariable predictors, obtained an area under the receiver operating characteristics curve of 0.901. Our model improves the reliability of simple clinical variables detected at the admission of the post-acute phase in predicting decannulation of ABI patients, thus helping clinicians to plan better rehabilitation.

## 1. Introduction

In patients with severe acquired brain injury (ABI), a tracheostomy is usually performed during the first days after the acute event when there is a need for prolonged mechanical ventilation and airway protection in the intensive care unit (ICU) [1]. However, in the transition from the intensive medical unit to the intensive rehabilitation unit (IRU), tracheal cannula continues to be present in a large amount of ABI patients [2]. Long-term tracheostomy may increase the risk of adverse effects such as respiratory infections and airway obstructions, thus limiting patients’ participation in the rehabilitation process [3,4]. Therefore, decannulation has the potential to improve care management, decrease morbidity, and increase the quality of life of post-acute ABI patients [4,5,6,7].

The early identification of patients who are eligible for decannulation is a common milestone of brain injury research. Several predictors of decannulation have been described in the literature. Good management of secretions and reactive cough is generally agreed to be a key factor for safe decannulation [8,9,10]. Prognostic models have been developed to predict the probability of decannulation using routinely gathered clinical data. Mortensen et al. [11] found that the strongest predictors for decannulation were age and a combination of overall functional abilities (measured with the early functional abilities score) combined with swallowing ability. In a review, Santus et al. [12] proposed a mixed combination of quantitative (cough, tube capping ≥ 24 h) and semi-quantitative parameters (level of consciousness, secretion, swallowing, age <70, indication for tracheostomy, comorbidities) for determining the probability of decannulation. Using a logistic regression model, Heidler et al. [13] found three particular clinical factors strictly associated with the probability of decannulation in 831 tracheotomized and weaned patients: age, prolonged duration of mechanical ventilation, and medical complications. Finally, in 2018, Reverberi et al. [7] published the DecaPreT score, a multivariable equation to estimate the probability of safe decannulation. The set of variables included in this tool were age, the pathogenesis of ABI, saliva aspiration, voluntary and reflex cough, and the level of consciousness. They demonstrated, in a relatively large population of patients with severe dysphagia secondary to ABI, that DecaPreT predicts effective decannulation with an accuracy of 83%.

However, other clinical variables have not been included in these models and need to be evaluated. For instance, clinical data such as coma recovery scale-revised (CRS-r) or length of stay in ICU could be reliable prognostic factors to better estimate the probability of decannulation in ABI patients [7]. Our aim was thus to validate a new set of clinical variables, on the basis of DecaPreT scores, to improve the prediction of decannulation in ABI patients during the sub-acute rehabilitation period.

## 2. Materials and Methods

### 2.1. Participants

All patients were consecutively admitted to the IRU of the Institute S. Anna (Crotone, Italy) between January 2016 and December 2020. Our unit manages patients with disorders of consciousness who have been directly transferred from critical care or acute neurosurgical units. From an initial cohort of 353 ABI patients, we enrolled only those who fulfilled the following inclusion criteria: (1) age ≥ 18 years; (2) presence of tracheostomy cannula on hospital admission; (3) severe ABI with Glasgow Coma Scale (GCS) ≤ 8 as measured at intensive care unit (ICU) discharge, identified based on ICU medical records relating to to the acute phase; (4) first admission to the neurorehabilitation unit. Exclusion criteria were (1) premorbid history of psychiatric disease or severe disability and (2) ICU length of stay > 90 days. Following inclusion and exclusion criteria, 75 ABI patients were excluded.

All patients were transferred directly from the ICU after the medical and neurosurgical complications had stabilized. Data from the acute hospital ICU were retrieved from patient files. The study was approved by the Ethical Committee of the Central Area Regione Calabria of Catanzaro (Protocol n. 244), according to the Helsinki Declaration. Written informed consent was obtained from the legal guardians of all the patients.

### 2.2. Data Collection

At the time of hospitalization, each patient was evaluated by a neuropsychologist with experience in evaluating disorders of consciousness with the use of CRS-r. All patients at admission were transferred from ICU or neurosurgery wards with a cuffed tracheostomy tube. Demographic data, the date of brain injury, ABI pathogenesis (classified trauma, hemorrhagic or ischemic vascular, hypoxia–anoxia, etc.), and the classification of consciousness disorder were recorded, together with voluntary and reflex cough and saliva aspiration. Saliva suction was evaluated using the blue dye test. The level of functional oral intake was assessed at the time of admission with the Italian version of the FOIS [14]. The endpoint of the study was safe decannulation defined as decannulation not followed by aspiration or need for a new application of tracheal cannula within 48–96 h.

### 2.3. Prediction Model and Statistical Analysis

Statistical analyses were performed using R, version 4.0.3 [15]. All data are presented as mean ± SD for normally distributed continuous variables, median (IQR) for nonnormal distributed continuous variables, or count (%) for categorical variables.

To validate the DecaPreT model, a two-steps approach was used. The update step followed the closed testing procedure presented in Vergouwe et al. [16]. Four logistic models were developed: (i) the “Original” model with the original regression coefficients; (ii) the “Calibration in the large” model with the intercept as the only free parameter; (iii) the “Logistic calibration” model, where both intercept and slope were recalibrated; (iv) the “Revision” model, where all the coefficients of the original predictors were reestimated. Then, a series of likelihood ratio tests of (i), (ii), and (iii) versus model (iv) were performed. Basing on the test results, one of the four logistic regression models was selected for the next step. Some of the tools used for this phase were downloaded from Darren L Dahly’s website and adapted to our analyses [17].

To test for possible refinement of the DecaPreT model further, new or modified predictors were included (age at injury in years considered as a continuous variable, CRS-r at admission and length of stay at ICU in days,) and eventual improvement in the performance with respect to the updated model analyzed. These reclassification models were evaluated using different classes of metrics: accuracy by Brier score, discrimination by the area under the ROC curve (AUC), calibration by calibration plot, E_max_ [18], and integrated calibration index (ICI) [19], and reclassification by continuous net reclassification index (NRI>0) and integral discrimination index (IDI). All the metrics were corrected for optimism and 95%CI was calculated using a bootstrap procedure (500 bootstrap samples).

## 3. Results

### 3.1. Clinical Characteristics at Admission and Discharge

From the initial cohort, 278 patients were selected for a retrospective observational evaluation of the rehabilitation program. During rehabilitation, five patients died and were discharged from the analysis. Clinical characteristics are reported in Table 1. The median sum of the DecaPreT score was 5 (IQR 3–7), and the median DecaPreT (estimated probability of decannulation) was 0.61 (0.29–0.82). A total of 61.5% of patients were safely decannulated before discharge.

**Figure 1:**
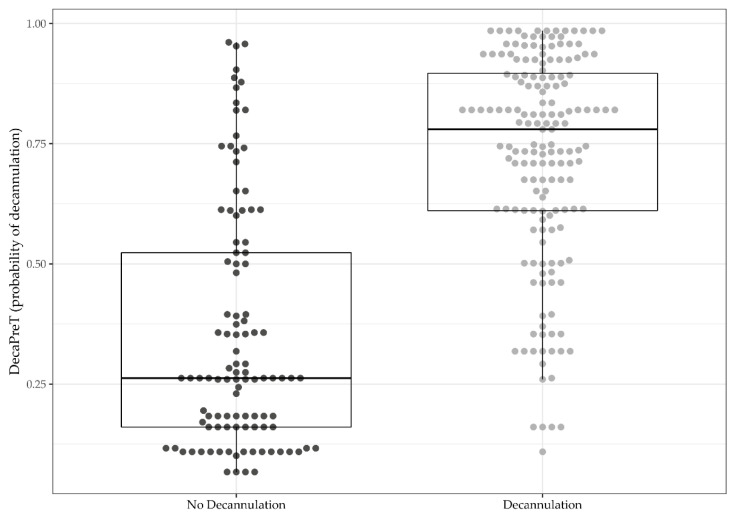
Distribution of probability of decannulation calculated by original DecaPreT equation between decannulated and not decannulated patients in our cohort.

Figure 1 shows the distribution of DecaPreT in decannulated (0.78, 0.61–0.90) and not decannulated (0.26, 0.16–0.52) patients.

### 3.2. Update of the DecaPreT Model

Table 2 shows the results of the closed testing procedure to select the updated model. All the tests were statistically significant (*p* < 0.0001); thus, the *Revision model* was adopted for further validation steps.

**Figure 2:**
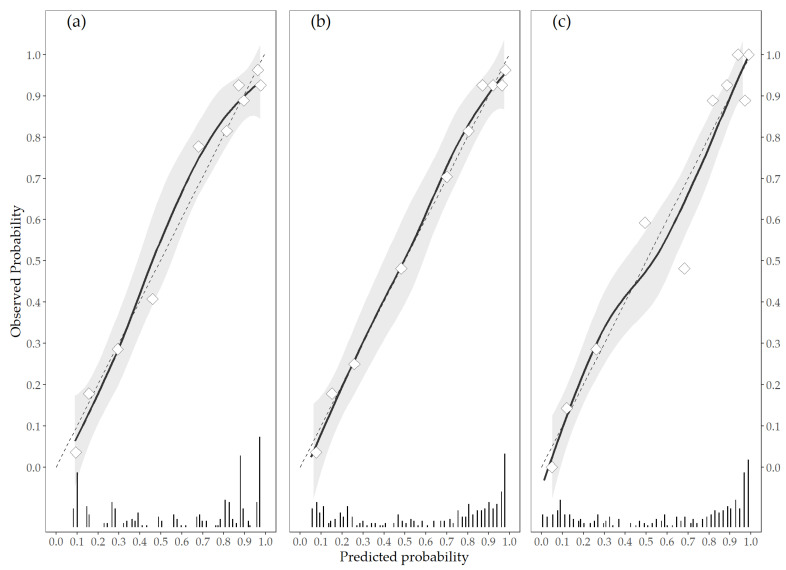
Calibration plots of predicted vs. observed probability in the Revision (**a**), Continuous (**b**), and Complete (**c**) models. The triangles represent deciles of subjects grouped by similar predicted risk; dashed line represents the linear calibration curve; solid line and grey area represent loess calibration curve and its standard error. The distribution of subjects by predicted probability is shown with bars at the bottom of the plot.

In order to improve the prediction of decannulation for ABI patients, two new logistic models with other predictors were fitted. Firstly, a Continuous model was developed replacing the DecaPreT Age and VS status scores with the age at injury in years and the CRS-r scores measured at IRU admission. Then, a Complete model was fitted adding the length of stay in ICU in days to the Continuous model (Appendix A—Table A1). Table 3 shows the metrics for the Revision, Continuous, and Complete models. The new logistic models showed a slightly better performance than the updated DecaPreT model. The Complete model had a better accuracy (Brier score 0.122, 0.093–0.146) and discrimination metrics (AUC 0.901, 0.866–0.939), while the Continuous model showed better calibration (E_max_ 0.018, 0.010–0.020; Figure 2). Both new models showed significantly better classification metrics than the revised DecaPreT model.

## 4. Discussion

In our study, we identified new predictors of decannulation in ABI patients. To do this, we combined a series of clinical variables into a multimodal model that estimates the probability of safe decannulation with an AUC of about 90%. To the best of our knowledge, this level of accuracy is the highest reported in any prognostic decannulation models, as compared to the 83% AUC by Reverberi et al. [7] and the 72% accuracy by Crary et al. [20]. Our small set of predictors included simple variables, such as CRS-r values and length of stay in ICU, which can be easily recorded by the neurorehabilitation team in any neurorehabilitation unit.

The inclusion of CRS-r scores in our model was already suggested by Reverberi et al. [7], who did not include this variable in their DecaPret model. These authors affirmed that a more accurate assessment of responsiveness and level of consciousness could better predict the probability of decannulation in ABI patients. This evidence was recently confirmed by Hikiki et al. [21], demonstrating that the highest CRS-r scores at admission corresponded to the highest probability of decannulation at discharge.

Similarly, the inclusion of the length of ICU as a predictor variable is an important clinical indicator, as already highlighted by Mortensen et al. [11]. Zivi et al. [22] also confirmed the strict relationship between lower ICU stay and the probability of decannulation, demonstrating that an early neurorehabilitation protocol carried out during the ICU period could reduce the time required for decannulation during the IRU period by around 30% (61 days against 94.5), compared to patients who started treatments at the neurorehabilitation ward.

Finally, we also included a new reevaluation of age at injury as a prognostic factor. This variable has been included in almost all previous prognostic models [20,23] since younger patients are known to generally have the highest probability of decannulation [7,18]. However, Reverberi et al. [7] modeled age as an ordinal variable, with scores of 0, 1, and 2 assigned to participants based on tertiles of the distribution of age in their sample. Instead, Mortensen et al. [11] considered age as a range of values (< 18 y, 18–40 y, 41–65 y, and > 65 y). Finally, Santus et al. [12] proposed a dichotomic division around the cutoff value of 70 years. In our model, age was considered as a continuous variable since this approach ensures the independence of the models from data distribution and, at the same time, avoids large changes in the estimated probability between age values that are close but included in different score categories. Moreover, the categorization of variables that are continuous in nature can lead to a loss of information, lower statistical power, and lower reliability [23,24,25].

Although our study was based on a retrospective analysis of data collected in a single center, only a few potentially eligible patients were not enrolled, and the assessment was conducted according to a rigorous, standardized protocol; therefore, selection and information biases are unlikely. Another possible limitation is that the validation sample used in the study was small. However, in order to reduce the risk of model overfitting and improve its generalizability, we used bootstrap procedures to calculate metrics and levels of optimism.

## 5. Conclusions

Our study provides an external means of validation and calibration of the DecaPret prediction model purely based on clinical data that can easily be obtained at the bedside. We identified new variables that estimate the probability of decannulation in ABI patients with a high level of accuracy. This assessment can be applied by trained personnel in a few minutes, thus contributing to better clinical management and a more favorable outcome. Further studies are needed to validate our model and evaluate how respiratory rehabilitation protocols might improve decannulation and which clinical factors (i.e., the amount of saliva above the cuff) might predict this short-term favorable outcome.

## Figures and Tables

**Table 1:** Clinical characteristics of the study cohort. Data are presented as mean ± SD for normally distributed continuous variables, median (IQR) for nonnormal distributed continuous variables, or count (%) for categorical variables.

	ABI (n = 273)
Age at injury (years)	53.01 ± 17.75
Female	93 (34.07)
Length of stay ICU (days)	36.00 (28.00–49.00)
CRS-r at admission	11.00 (5.00–21.00)
*Feeding at admission*	
Oral	12 (4.40)
NG tube	158 (57.88)
PEG	103 (37.73)
*Brainstem injury (%)*	43 (15.75)
*Age DecaPreT score*	
<47	98 (35.90)
47–61	86 (31.50)
>61	89 (32.60)
*Saliva Aspiration DecaPreT score*	
No	191 (69.96)
Yes	82 (30.04)
*Vegetative status DecaPreT score*	
No	168 (61.54)
Yes	105 (38.46)
*Coughing Score DecaPreT score*	
Voluntary and reflex	111 (40.66)
Reflex only	46 (16.85)
Voluntary only	50 (18.32)
Neither	66 (24.18)
Pathogenesis of brain lesion DecaPreT score	
Trauma	96 (35.16)
Other	15 (5.49)
Stroke	140 (51.28)
Anoxia	22 (8.06)
DecaPreT (probability of decannulation)	0.61 (0.29–0.82)

ABI: acquired brain injury; CRS-R: coma recovery scale-revise; ICU: intensive care unit; NG: nasogastric; PEG: percutaneous endoscopic gastrostomy.

**Table 2:** Likelihood ratio test for Original, Calibration at large, Logistic calibration vs. the Revision models.

Tested Models	Degrees of Freedom	χ^2^	*p*-Value
Revision vs. Original	6	36.6209	<0.001
Revision vs. Calibration in the large	5	34.3932	<0.001
Revision vs. Logistic calibration	4	34.3809	<0.001

**Table 3:** Performance metrics of the developed models.

Metric	Revision Model	Continuous Model	Complete Model
Brier score	0.134 (0.102–0.155)	0.130 (0.099–0.152)	0.122 (0.093–0.146)
AUC	0.874 (0.839–0.917)	0.884 (0.855–0.932)	0.901 (0.866–0.939)
Intercept	0.021 (−0.001–0.033)	0.019 (−0.004–0.036)	0.023 (−0.010–0.044)
Slope	0.943 (0.924–0.958)	0.935 (0.930–0.965)	0.933 (0.905–0.944)
E_max_	0.016 (0.011–0.022)	0.018 (0.010–0.020)	0.019 (0.014–0.028)
ICI	0.041 (0.020–0.071)	0.023 (0.016–0.063)	0.018 (0.007–0.053)
NRI (>0) *		0.545 (0.314–0.776)	0.724 (0.497–0.950)
IDI *		0.021 (−0.001–0.044)	0.057 (0.025–0.089)

* vs. Revision model. ICI: integrated calibration index; NRI: net reclassification index; IDI: integrated reclassification index.

## Data Availability

The data presented in this study are available on request from the corresponding author. The data are not publicly available due to privacy restrictions.

## Appendix A

**Table A1:** Models estimated coefficients.

	Estimate	SE	*p*-Value
**Revision model**			
Intercept	−2.452	0.43	<0.0001
Age score	0.056	0.222	0.8
Saliva score	0.137	0.458	0.766
VS score	1.718	0.392	<0.0001
Coughing score	1.212	0.174	<0.0001
Pathogenesis score	0.23	0.164	0.161
**Continuous model**			
Intercept	−2.613	0.868	0.003
Age (years)	−0.01	0.011	0.393
Saliva score	0.154	0.47	0.743
CRS-r	0.129	0.025	<0.0001
Coughing score	1.123	0.173	<0.0001
Pathogenesis score	0.18	0.173	0.297
**Complete model**			
Intercept	−0.822	0.976	0.4
Age (years)	−0.018	0.012	0.135
Saliva score	0.161	0.478	0.737
CRS-r	0.13	0.026	<0.0001
Coughing score	1.146	0.181	<0.0001
Pathogenesis score	0.151	0.179	0.399
Length of stay in ICU (days)	0.13	0.026	<0.0001

VS: Vegetative status; CRS-r: coma recovery scale-revised; ICU: intensive care unit.

DecaPret update equations:

(A1) $$\mathrm{Continuous}\;\mathrm{Model}=\frac{1}{1+e^{-\left(-0.01\times Age+0.154\times Saliva\;Score+0.129\times CRS-r+1.123\times Coughing\;Score+0.18\times Pathogenesis\;Score-2.613\right)}}$$

(A2) $$\mathrm{Complete}\;\mathrm{Model}=\frac{1}{1+e^{-\left(-0.018\times Age+0.161\times Saliva\;Score+0.13\times CRS-r+1.146\times Coughing\;Score+0.151\times Pathogenesis\;Score+0.13\times Days\;in\;ICU-0.822\right)}}$$
